# Predicting Hepatocellular Carcinoma With Minimal Features From Electronic Health Records: Development of a Deep Learning Model

**DOI:** 10.2196/19812

**Published:** 2021-10-28

**Authors:** Chia-Wei Liang, Hsuan-Chia Yang, Md Mohaimenul Islam, Phung Anh Alex Nguyen, Yi-Ting Feng, Ze Yu Hou, Chih-Wei Huang, Tahmina Nasrin Poly, Yu-Chuan Jack Li

**Affiliations:** 1 Taipei Medical University Taipei Taiwan

**Keywords:** hepatocellular carcinoma, deep learning, risk prediction, convolution neural network, deep learning model, hepatoma

## Abstract

**Background:**

Hepatocellular carcinoma (HCC), usually known as hepatoma, is the third leading cause of cancer mortality globally. Early detection of HCC helps in its treatment and increases survival rates.

**Objective:**

The aim of this study is to develop a deep learning model, using the trend and severity of each medical event from the electronic health record to accurately predict the patients who will be diagnosed with HCC in 1 year.

**Methods:**

Patients with HCC were screened out from the National Health Insurance Research Database of Taiwan between 1999 and 2013. To be included, the patients with HCC had to register as patients with cancer in the catastrophic illness file and had to be diagnosed as a patient with HCC in an inpatient admission. The control cases (non-HCC patients) were randomly sampled from the same database. We used age, gender, diagnosis code, drug code, and time information as the input variables of a convolution neural network model to predict those patients with HCC. We also inspected the highly weighted variables in the model and compared them to their odds ratio at HCC to understand how the predictive model works

**Results:**

We included 47,945 individuals, 9553 of whom were patients with HCC. The area under the receiver operating curve (AUROC) of the model for predicting HCC risk 1 year in advance was 0.94 (95% CI 0.937-0.943), with a sensitivity of 0.869 and a specificity 0.865. The AUROC for predicting HCC patients 7 days, 6 months, 1 year, 2 years, and 3 years early were 0.96, 0.94, 0.94, 0.91, and 0.91, respectively.

**Conclusions:**

The findings of this study show that the convolutional neural network model has immense potential to predict the risk of HCC 1 year in advance with minimal features available in the electronic health records.

## Introduction

Liver cancer is the sixth most cancer in incidence and the fourth leading cause of cancer-related mortality worldwide [1]. The most common type of liver cancer is hepatocellular carcinoma (HCC), accounting for approximately 80% of all liver cancer [1]. The incidence and mortality rate of HCC are higher in Sub-Saharan Africa and Southeast Asia than in the United States [2]. HCC incidence has been increasing globally, including in the USA, and is expected to continue growing over the next 20 years due to the higher number of patients with advanced hepatitis C virus and nonalcoholic steatohepatitis [3,4]. A significant number of studies (epidemiological and clinical) have reported risk factors of HCC that can be used to correctly stratify patients at risk and to implement prevention measures [5,6]. Accurate risk stratification tools may contribute to the timely identification of HCC patients and facilitate early detection and diagnosis.

The recent widespread adaption of electronic health records (EHRs) has caused the proliferation of clinical data and offers tremendous potential for predicting different diseases early, including cancer [7,8]. The use of EHRs can also contribute to high-quality treatment, improved patient management, reduced health care costs, and efficient clinical research [9,10]. Multiple studies have demonstrated that risk prediction models can anticipate the future incidence of HCC and ensure early treatment [8,11]. Flemming et al [12] recently developed a model for predicting the 1-year risk of HCC among patients with cirrhosis, but the performance was not satisfactory.

Convolutional neural network (CNN) models have already shown remarkable performance in detecting diseases from digital images and predicting diseases from EHRs [13]. CNN models take advantage of the hierarchical pattern in EHRs and assemble more complex patterns using smaller and simpler patterns. Thus far, however, no study has used deep learning algorithms, including CNN models, to predict HCC. Therefore, we developed a CNN model that analyzes EHRs to accurately predict HCC risk. We presented each patient’s EHR data as a matrix which was formed by the medical events versus the temporal continuity and regarded the matrix as a 2D EHR image. With the time information, the EHR image revealed the severity and the trend of the medical events explicitly, which were beneficial to HCC risk classification.

## Methods

### Data Sources

We collected data from Taiwanese National Health Insurance Research Database, a rich source of data with the medical histories of 23 million people (approximately 99.9% of the total population in Taiwan). The database contains demographic, medication (number of prescriptions, the brand and generic name of the drugs, the date of the prescriptions, the dosage of the medication), and diagnostic information. The database is of excellent quality and completeness, and is used to conduct high-quality research. The Taipei Medical University research ethical board approved this study. Participant consent was not required because all individual’s information was deidentified.

### Study Population

We screened HCC cases and their information from a subset of 2 million patients from the National Health Insurance Research Database of Taiwan from January 1, 1999, to December 31, 2013. We also randomly sampled non-HCC patients, and there were nearly 4 times the number of non-HCC cases as there were HCC cases from the same database. We chose this multiple because the increase of predictive performance slowed down after a control:case ratio beyond 4 to 5 in our experiment and another study [14]. Moreover, all the participants were between 20 and 90 years old.

### HCC Patients

HCC cases were identified by the International Classification of Disease, Ninth Revision, clinical modification (ICD-9-CM) code 155. HCC patients were ascertained only when they also met one of the following criteria: individuals registered as having cancer in catastrophic illness file, individuals with a primary cancer diagnosis in inpatient admission, and individuals that took HCC treatment medications or any specific procedure for HCC.

### Variables Employed

The input variables for the predictive model included deidentified patient’s ID, gender, age, diagnosis code, visiting date, prescription code, and exposure time of drugs. However, only the first 3 digits of the ICD-9-CM were adopted to represent the disease information. After 88 undefined codes were excluded, 993 ICD-9-CM were considered in this study, including V-code (Multimedia Appendix 1). Drug exposure was reflected by the World Health Organization Anatomical Therapeutic Chemical (ATC) classification system. We took the first 5 characters to cover most drugs in the same category; for example, the 5-digit ATC code (C09AA = angiotensin-converting enzyme inhibitors, plain) included all plain angiotensin-converting enzyme inhibitors, such as C09AA01 (captopril), C09AA02 (enalapril), and so forth. Nevertheless, 7 characters (eg, R06AX12) were considered for the other drugs with “X” as the fifth character because usually “X” means other agents in ATC code. There were 699 ATC codes expressed in this manner among these enrolled patients.

### Constructing the EHR Image

**Figure 1:**
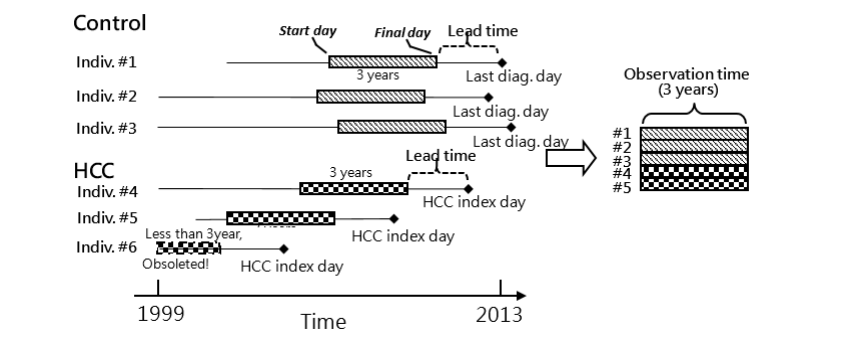
Preprocess from electronic health record to matrix. Diag: diagnosis: HCC: hepatocellular carcinoma; Indiv: individual.

Three-year (observation time) data of every enrolled patient were extracted from the National Health Insurance Research Database. To predict HCC's risk 1 year in advance, the final day of the extracted data was 1 year (advanced time) before the index day, as shown in Figure 1. For patients with HCC, the index day was the day they were diagnosed with HCC, while that of the non-HCC patients was the last day they had a diagnosis code in the data set. We chose 3 years as the observation time as a trade-off: the longer the observation time, the fewer the number of eligible patients with a sufficient data period there would be; on the other hand, with a shorter time window, the amount of data of each patient would be less. We needed a total 4 years of data for every patient, including 3 years of training and the skipping of the last year. In other words, 3 years of data were used to predict the next year of HCC cancer risk. Another thing to remember is that the duration of the drug exposure was not counted repeatedly if the times of the drug orders overlapped in different prescriptions.

**Figure 2:**
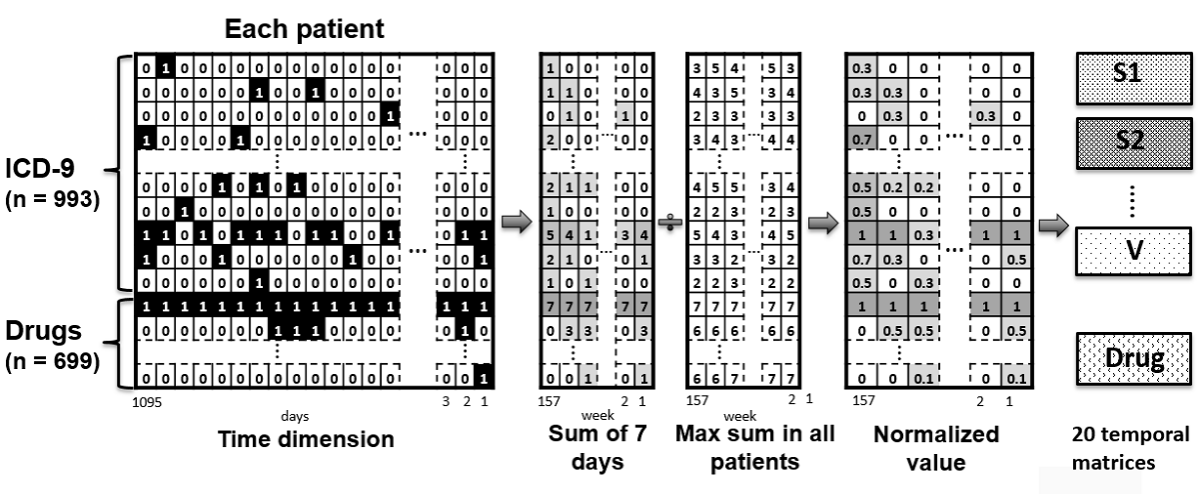
Preprocess from the matrix to 20 electronic health record images. ICD-9: International Classification of Disease, Ninth Revision; S1: subgroup 1 (001-139) of ICD-9; S2: subgroup 2 (140-239) of ICD-9; V: V codes, a supplementary classification in ICD-9.

We used these extracted data to construct the matrices which were regarded as the EHR images for each patient and which were afterward used to train and validate the CNN model for HCC risk prediction. The rows of the matrices were the diagnostic codes and the drug codes, and the columns were the temporal information of those events. Once the patient was diagnosed with a certain ICD-9-CM code or given a certain ATC code on a certain day, “1” was assigned to the corresponding coordinate of his or her matrix. At the end, to reduce the column size of the matrix, we rounded up the temporal coordinate by a period of 7 days, meaning the unit of the temporal sequence was 1 week instead of 1 day after being aggregated. Furthermore, to normalize the sum value (0 to 7) of the elements in the matrix to 0-1 for the following CNN computation, each element was divided by the maximum value of all the enrolled patients at the same coordinate. Considering it is not reasonable to mix different organ systems with a common CNN filter, we broke the ICD-9-CM down into 19 organ systems. Adding up the drug group, we found a total of 20 images for each patient to develop the deep learning model, as shown in Figure 2.

### Architecture of the CNN Model

CNN is a biologically inspired variant of a multi-layer perceptron, which uses filters to extract the features of the input by dot production [15,16]. We applied 5 hidden layers between the input and the output layer. Among them, the first one was a convolution layer with 4 filters in the shape of 1 × 57, where 157 was the number of the weeks in 3 years and the number of columns of the input matrix. The filters were trained to learn the weighting of the temporal sequence of each organ system and the drug group.

**Figure 3:**
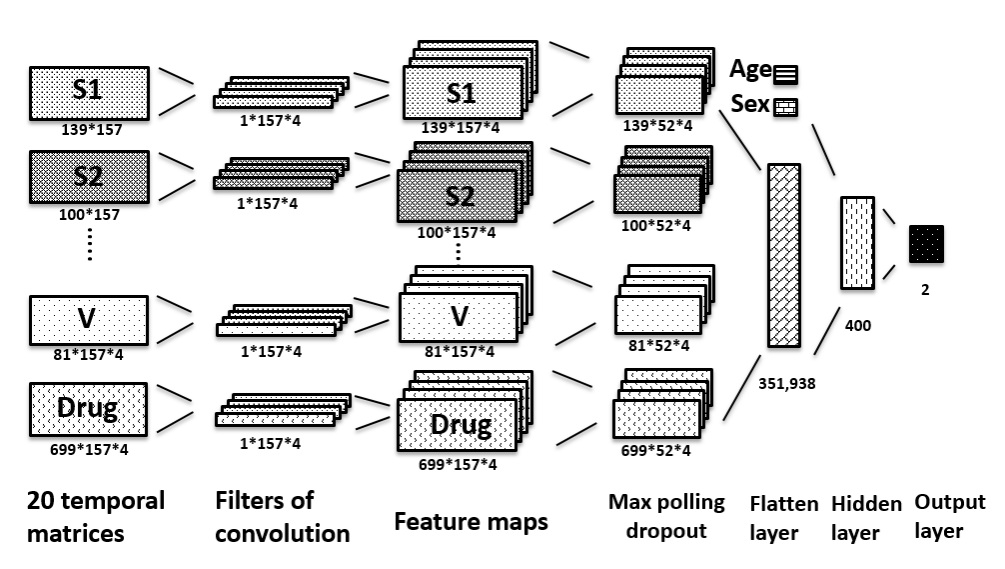
Structure of the convolutional neural network. S1: subgroup 1 (001-139) of the International Classification of Disease, Ninth Revision (ICD-9); S2: subgroup 2 (140-239) of ICD-9; V: V codes, a supplementary classification in ICD-9.

The second layer was a max-pooling layer with a size of 1 × 3 to reduce the sparsity of the learned features and was followed by a dropout layer that set 10% of the data to 0 at random to prevent the overfitting of the model. The fourth layer flattened the output of the previous layer and concatenated age and gender information. The fifth layer was a fully connected layer with 400 neurons. Finally, the output layer had 2 neurons, representing high risk and low risk, with the softmax classifiers to indicate the predictive result, as shown in Figure 3.

As for the hyper-parameters of the CNN model, the epoch was set as 2 to obtain the optimal area under the receiver operating curve (AUROC) according to our experimental result. The batch size was 32, and the learning rate was optimized by the AdaDelta method [17]. Moreover, the activation function used in the first 3 layers was the rectified linear unit [16]. To eliminate the bias of data sampling, we introduced 5-fold cross-validation [18] to evaluate the performance of this model. Therefore, each time, 80% of all patients were applied for training and the remaining 20% were used for validation by turn. The final performance was assessed by the average of all AUROC of the 5 folds.

### Statistical Analysis

In this study, continuous numeric variables are presented by mean and SD, while the categorical variables are described by frequency and percentage. The performance of the model was assessed by the AUROC, sensitivity, and specificity. Moreover, we used odds ratio (OR) as an indicator to compare to the weighting of the variables in the CNN model to check their consistency. OR is a statistic that quantifies the strength of the association between 2 events, which in this study were ICD-9-CM (or ATC code) and HCC. If the OR is greater than 1, then the 2 events are considered to be associated. Conversely, if the OR is less than 1, they are considered to be negatively correlated. For the calculation of the OR, the ICD-9-CM or ATC code was considered as true only when they occurred 3 times or more in the extracted 3 years of EHR data. In stepwise fashion, we set the content of each input variable to 0 and checked the AUROC loss against the result of the full input. The variable would have higher weighting if it underwent more AUROC loss in the testing like the feature selection [19].

All analyses were performed using R language (The R Foundation for Statistical Computing). Keras, a high-level neural network application programming interface was applied as the top of TensorFlow to construct the mentioned CNN model in this study. Running on a computer with Intel i7 CPU, 64GB DRAM, and an Nvidia GTX 1080 GPU with 8GB DRAM, the 5-fold cross-validation took 80 minutes to complete.

## Results

A total of 47,945 patients (24,664 males and 23,281 females) were included in this study, with 9553 being diagnosed with HCC and 38,392 being non-HCC patients. The mean age of HCC patients was 59.9 (SD 14) years while that of the control patients was 47.5 (SD 17.3) years. Moreover, the portion of the male patients in the HCC group and the control group was 64.64% (6175/9553) and 48.16% (18,489/38,392), respectively. Table 1 shows the demographic variables of the HCC and control groups.

The overall AUROC of predicting HCC patients 1 year in advance was 0.94 (95% CI 0.93-0.94), with a sensitivity of 0.869 and a specificity of 0.865. The threshold for the output of the CNN model to classify the risk group was 0.11, which was chosen by the maximum sum value of the sensitivity and the specificity. We also evaluated the performance of the model with different advance times. The overall AUROC when predicting HCC patients at 7 days, 6 months, 1 year, 2 years, and 3 years early was 0.96, 0.94, 0.94, 0.91, and 0.91, respectively.

Furthermore, different input groups and their combination were applied separately to assess their value. Our 1-year-in-advance predictive model with training and validating completed with only age and gender information achieved an AUROC of 0.73. The AUROC was 0.86 when only the disease codes were used and 0.88 when age, gender, and the disease codes were used. Meanwhile, the model applying only ATC achieved an AUROC of 0.91, while the application of age, gender, and ATC yielded an AUROC of 0.92.

**Table 1:** Demographics of the sampled data set.

Demographic			HCC (n=9553)		Control (n=38,392)		Difference
Age (years), mean (SD)			59.9 (14)		47.5 (17.3)		N/A
Male, n (%)			6175 (64.6)		18489 (48.2)		N/A
**ICD-9^a^, mean diagnoses per patient in 3 years (ordered by difference)**							
	Total	126.2		78.1		48.1	
	571 (Chronic liver disease and cirrhosis)	8.36		0.61		7.75	
	250 (diabetes mellitus)	6.23		2.27		3.96	
	070 (viral hepatitis)	3.19		0.3		2.89	
	401 (essential hypertension)	5.43		2.83		2.59	
	465 (acute upper respiratory infections)	5.37		3.63		1.73	
	780 (general symptoms)	4.16		2.43		1.73	
	533 (peptic ulcer)	1.93		0.55		1.38	
	372 (disorders of conjunctiva)	2.78		1.59		1.19	
	724 (disorders of back)	2.38		1.22		1.17	
	402 (hypertensive heart disease)	1.89		0.76		1.12	
**ATC^b^ code, mean prescribed days per patient in 3 years (ordered by difference)**							
	Total	2362		1099		1263	
	A05BA (liver therapy)	97.87		5.8		92.08	
	A10BB (blood glucose–lowering drugs)	93.51		29.88		63.63	
	C08CA (selective calcium channel blockers with vascular effects)	119.37		60.93		58.44	
	A10BA (blood glucose–lowering drugs)	77.87		32.36		45.51	
	B01AC (platelet aggregation inhibitors)	87.12		45.71		41.42	
	N05BA (benzodiazepine, for anxiolytics)	71.18		34.29		36.89	
	A02AX (antacids for acid related disorders)	42.61		14.68		27.93	
	C07AB (beta=blocking agents, cardiovascular system)	58.82		31.39		27.43	
	C09AA (ACE^c^ inhibitors, cardiovascular system)	42.13		15.36		26.77	
	A02AF (antacids with antiflatulents)	27.91		2.06		25.84	

^a^ICD-9: International Classification of Disease, Ninth Revision.

^b^ATC: Anatomical Therapeutic Chemical (classification system).

^c^ACE: angiotensin-converting enzyme.

Table 2 shows the AUROC impact of age, gender, and some diseases when they were withdrawn from the model, together with their ORs, against HCC. Some high impact variables were chronic liver disease and cirrhosis (AUROC loss 2.52%), viral hepatitis (0.67%), age (0.57%), peptic ulcer (0.41%), gender (0.39%), and screening for malignant neoplasms (0.78%), all of which were negatively associated with HCC due to having an OR of less than 1. Table 2 also shows some variables with extremely high or low ORs, but their AUROC was not high because the number of patients was not large; these variables included varicose veins (OR 22.47), other disorders of the liver (OR 5.34), normal pregnancy (OR 0.16), and others. Table 2 also shows the ORs of a cohort whose age and gender were matched with those of the HCC cohort, and their individual number was also 4 times greater than that of the HCC cohort, which was similar to the random sampled cohort. After the correlation of age and gender with HCC was decoupled, the ORs of the matched cohort did not appear to be as critical as those of the random sampled cohort, but their trends were consistent.

**Table 2:** Age, gender, and diseases with AUROC loss greater than 0.01% or OR greater than 4 or less than 0.3.

Characteristic		AUROC^a^ loss (%) (95% CI)	OR^b^ (95% CI)	Patient number	Age- and gender-matched OR (95% CI)
	Age > 50 years	0.57 (0.6-0.54)	4.26 (4.0-4.49)	24,074 (50.2)^c^	N/A^d^
	Male	0.39 (0.42-0.36)	1.97 (1.88-2.06)	24,671 (51.4)^c^	N/A
**ICD-9^e^ (description)**					
	571 (chronic liver disease and cirrhosis)	2.52 (2.35-2.62)	14.63 (13.75-15.58)	5753	11.08 (10.5-11.7)
	070 (viral hepatitis)	0.67 (0.58-0.77)	11.36 (10.48-12.32)	3023	8.98 (8.4-9.6)
	533 (peptic ulcer)	0.41 (0.69-0.13)	3.46 (3.22-3.72)	3446	2.92 (2.7-3.1)
	456 (varicose veins of other sites)	<0.01	22.47 (15.89-31.78)	246	14.31 (11.3-18.1)
	573 (other disorders of liver)	<0.01	5.34 (4.54-6.27)	611	3.93 (3.5-4.4)
	794 (nonspecific abnormal results of function studies)	<0.01	4.48 (3.59-5.6)	313	3.03 (2.6-3.6)
	574 (cholelithiasis)	<0.01	4.36 (3.75-5.06)	700	3.9 (3.4-4.4)
	V76 (screening for malignant neoplasms)	0.78 (1.2-0.36)	0.42 (0.36-0.48)	2445	0.42 (0.4-0.5)
	626 (disorders of menstruation and other abnormal bleeding from female genital tract)	0.14 (0.31-0.01)	0.3 (0.27-0.35)	3352	0.66 (0.6-0.7)
	625 (pain and other symptoms associated with female genital organs)	0.16 (0.33-0.01)	0.25 (0.18-0.35)	674	0.57 (0.4-0.7)
	V22 (normal pregnancy)	<0.01	0.16 (0.12-0.22)	1180	0.44 (0.3-0.6)

^a^AUROC: area under the receiver operating curve.

^b^OR: odds ratio.

^c^These data are presented as numbers and percentages.

^d^N/A: not applicable.

^e^ICD-9: International Classification of Disease, Ninth Revision.

Table 3 displays the AUROC-impacted value and ORs of the drugs. The high impact drugs include liver therapy (AUROC loss 1.35%), antacids with antiflatulents (1.2%), solutions for parenteral nutrition (0.77%), aluminum compounds (0.63%), antihistamines (0.57%), and others. Some drugs appear to be negatively associated with HCC, including the treatment of acne (0.48%) and progestogens (0.36%), but this does not mean that they could reduce the risk of HCC since only an association, and not causation, was discovered between them. Given the age- and gender-matched cohort, the ORs greater than 1 could be considered similar to those of the unmatched cohort, while the ORs less than 1 were not so low.

**Figure 4:**
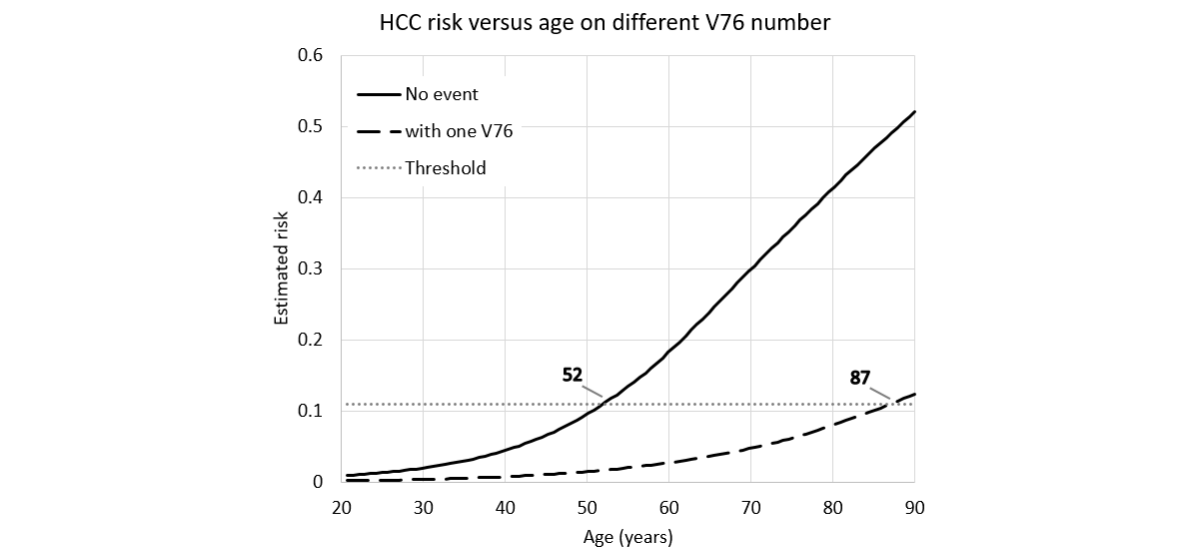
An example of a male patient. HCC: hepatocellular carcinoma.

We referred to this CNN predictive model while testing a special case in which a male patient had only age and gender information but did not have any medical records during the observed 3 years. The estimated HCC risks are listed in Figure 4 according to his age. In this case, the patient was classified into the high-risk group at the age of 52 years. However, if the patient had 1 record of screening for malignant neoplasms (V76 of the ICD-9-CM) a half year before the final day of his EHR and the result was benign, the high-risk alarm would be delayed until the age of 87 years. The reason for this is that the screening for malignant neoplasms was negatively relevant to HCC.

**Table 3:** Drugs with high AUROC-impacted value.

ATC^a^	AUROC^b^ loss (%) (95% CI)	OR^c^ (95% CI)	Patient number	Age- and gender-matched OR
A05BA (liver therapy)	1.35 (1.27-1.42)	14.26 (13.29-15.31)	4353	12.03 (11.3-12.8)
A02AF (antacids with antiflatulents)	1.2 (1.19-1.21)	10.38 (9.72-11.08)	4707	11.87 (11.1-12.7)
B05BA (solutions for parenteral nutrition)	0.77 (0.67-0.87)	5.18 (4.71-5.69)	1873	6.07 (5.5-6.7)
A02AB (aluminum compounds)	0.63 (0.56-0.76)	3.27 (3.04-3.51)	3335	3.31 (3.1-3.6)
R06AX12 (antihistamine, treatment of allergy)	0.57 (0.42-0.65)	31.78 (26.28-38.43)	1008	37.58 (31-45.6)
B05XC (vitamins)	0.56 (0.52-0.59)	5.92 (5.19-6.76)	942	6.38 (5.6-7.3)
A11JC (vitamins, other combinations)	0.5 (0.09-0.98)	13.68 (11.7-15.98)	890	14.46 (12.4-16.8)
C03DA (antimineralocorticoid)	0.45 (0.44-0.45)	6.42 (5.73-7.19)	1286	7.2 (6.4-8.1)
A05AA (bile therapy)	0.42 (0.27-0.51)	14.13 (12.28-16.25)	1115	11.39 (10.1-12.9)
A11BA (multivitamins)	0.39 (0.21-0.67)	7.47 (5.48-10.18)	176	7.78 (5.8-10.5)
A11AA (multivitamins with minerals)	0.36 (0.03-0.58)	7.7 (6.22-9.52)	379	6.92 (5.7-8.4)
B02BA (vitamin K)	0.25 (0.03-0.6)	7.58 (5.61-10.23)	189	6.53 (5-8.6)
D10AF (treatment of acne)	0.48 (0.15-1)	0.4 (0.36-0.46)	3017	0.72 (0.6-0.8)
G03DC (progestogens)	0.36 (0.08-0.53)	0.39 (0.33-0.46)	1809	0.91 (0.8-1.1)
D10AX03 (azelaic acid, antiacne)	0.31 (0.09-0.57)	0.36 (0.26-0.5)	503	0.72 (0.5-1)

^a^ATC: Anatomical Therapeutic Chemical (classification system).

^b^AUROC: area under the receiver operating curve.

^c^OR: odds ratio.

## Discussion

### Main Findings

Accurate stratification of patients at high risk for HCC is the primary step for early detection and treatment. Our predictive model, based on a CNN algorithm and using minimal features from electronic medical records, can correctly stratify HCC risk in patients. The main advantages of our model are that it can predict patients with HCC 6 months, 1 year, and 3 years early with an AUROC as high as 0.96, 0.94, and 0.91, respectively. Furthermore, this model does not require any laboratory data. It is entirely based on age, gender, diseases, and drug data from the EHR as part of routine patient care. Finally, results of the prediction are reliable and can be trusted; this paper presents the highly weighted variables and checked their OR against HCC to gain insight into the black box of the CNN model. HCC risk stratification performed 1 to 3 years in advance could help physicians in identifying the high-risk patients and thus improving treatment and surveillance in an evidence-based fashion, such as by actively treating hepatitis C, instructing patients to improve their lifestyle, or screening for malignant neoplasms before normally scheduled.

### Comparison With Other Studies

Several groups of researchers have already attempted to improve the identification and risk stratification of HCC patients. Flemming et al [12] showed that the ADRESS-HCC risk model (including 6 variables of age, diabetes, race, etiology of cirrhosis, sex, and severity of liver dysfunction) could identify HCC patients 1 year earlier with an ROC of 0.70. A total of 34,932 patients were included in their model, and the median follow-up was 1.26 years. The traditional statistical regression was used to develop and validate the predictive model for HCC risk. Furthermore, Yang et al [20] developed a predictive model of HCC risk of over 5 or 10 years in advance in patients with chronic hepatitis B. Potential risk factors, including age, sex, alcohol consumption, and serum alanine aminotransferase level, were considered to develop and validate the predictive model. The regression model achieved AUROCs ranging from 82.1% to 88.5%, and the nomograms model achieved AUROCs ranging from 82.1% to 86.6%. In comparison, our model can predict HCC risk 1 year ahead as opposed to a longer 5-10 year period; in this way, patients at high risk are more likely to undergo further medical treatment for the more immediate hazard instead of putting it off.

### Clinical Implications

This deep learning–based model works by analyzing the pattern relationships of existing data. The CNN model with multiple hidden layers has already shown remarkable success for image classification [21]. However, there is still no deep learning–based HCC risk predictive model that uses EHR data. As EHRs are a rich source of patient data, CNN models can organize these high-dimensional data sets to provide greater prediction for patients with HCC. Making use of artificial intelligence to facilitate HCC prediction is beneficial because current clinical guidelines indeed have little effect on predicting those patients with HCC 1 year earlier and usually require complementary laboratory data.

Preventing HCC is the main target in the care of a patient with multiple risk factors. A prevention strategy should focus on reducing the development of HCC risk factors or treating them in the early stage [22]. The best approaches in HCC prevention usually include identifying high-risk factors and eliminating these factors if possible. This study presents the diseases and the drugs with high weighting in the model as well as those with higher ORs. These have also been reported in other studies. A significant amount of literature has already indicated that age, gender [7], and diseases like viral hepatitis, peptic ulcer, chronic liver disease, and cirrhosis [23-25] are associated with the development of HCC. Also, some studies found evidence for a relation between vitamins and liver diseases such as fibrosis [26] or nonalcoholic fatty liver disease [27]. Mineralocorticoid receptor activation could play a role in hepatic fibrogenesis, and its modulation could be beneficial for nonalcoholic steatohepatitis [28]. Moreover, a liver drug, silymarin, has been used to good effect in different liver disorders due to its antioxidant, anti-inflammatory, and antifibrotic properties [29]. Previous studies have shown that the use of antacids promotes liver disease [30], and the high impact of antacids (see Table 3) should be further investigated to determine whether a causal relationship exists.

Another noteworthy finding was that some variables had high ORs for HCC but were not in the list of highly weighted variables. This may be because the number of the patients diagnosed with these variables was not large enough to garner heavy weighting. For example, ICD-9 code 456 (varicose veins of other sites) had an OR as high as 22.47, but the AUROC loss for it was less than 0.1% because there were only 246 patients with this code out of the 9553 patients with HCC and the total population of 47,945.

As correlation is not necessarily causation [31], it cannot be concluded that those variables with high ORs induce HCC: they are only positively correlated with it. However, these can still be considered significant variables and be used to predict HCC risk. For example, we cannot claim antacids with antiflatulents induce HCC despite their OR for HCC being as high as 10.38. However, the patients taking these drug do have a higher probability of having HCC due to its relationship with HCC.

On the other hand, OR of screening for malignant neoplasms was less than 0.5, which means it is negatively correlated with HCC. The reason for this correlation is that the neoplasms screening records of the patients with HCC do not increase after day they are diagnosed with HCC, while the non-HCC patients continuously accumulate screening records until the last day of their extracted data. Furthermore, the reason why some diagnoses in Table 2, including endometriosis, symptoms associated with female genital organs, and pregnancy, negatively correlate with HCC is that they are more commonly associated with young females who have the opposing traits to those considered as high-risk factors of HCC: being old and male. The inspection of the ORs corresponding to the highly weighted variables also helps us to understand how the predictive model works.

### Strengths and Limitations

Our model has several strengths. First, this is the first study to use a deep learning–based predictive model to stratify patients with HCC 1 year in advance via the claim database. Second, our study achieved a higher performance than did previous studies all while using a minimal number of features from standardized and widely available clinical data of EHRs. Despite the promising results in stratifying HCC patients, our study has several limitations that should be addressed. First, laboratory data inclusion may enable more accurate deep learning models to be trained and validated with higher confidence. Second, several variables such as genetic data, ethnicity, family history, alcohol consumption, smoking, dietary habit, vital signs, and BMI were not considered in our predictive model, the inclusion of which may improve the prediction; nonetheless, our model achieved a high performance with the currently available variables in EHRs. Finally, external validation on other data sets are warranted to the ensure generalizability of our current model.

### Conclusions

Our prediction model achieved high performance with high sensitivity and specificity for predicting HCC risk using standardized and widely available claim data. This predictive model also identified some risk factors and may provide physicians a means to recognizing deteriorating patients in timely fashion. As the model predicts patients with HCC 1 year in advance, it is therefore able to improve patient care and enhance research into best practices to further reduce mortality in patients with HCC.

## Appendix

Multimedia Appendix 1 Supplementary files.

## Abbreviations

ATC: Anatomical Therapeutic Chemical (classification system)
AUROC: area under the receiver operating curve
CNN: convolutional neural network
EHR: electronic health record
HCC: hepatocellular carcinoma
ICD-9-CM: International Classification of Disease, Ninth Revision, clinical modification
OR: odds ratio
