# Supplementary material for: Predicting Hepatocellular Carcinoma With Minimal Features From Electronic Health Records: Development of a Deep Learning Model
Source: JMIR Cancer. 2021 Oct 28;7(4):e19812. doi: 10.2196/19812 (PMC8587326; doi:10.2196/19812)
Supplement: Multimedia Appendix 1 [file cancer_v7i4e19812_app1.docx]

**Supplementary materials:**

**
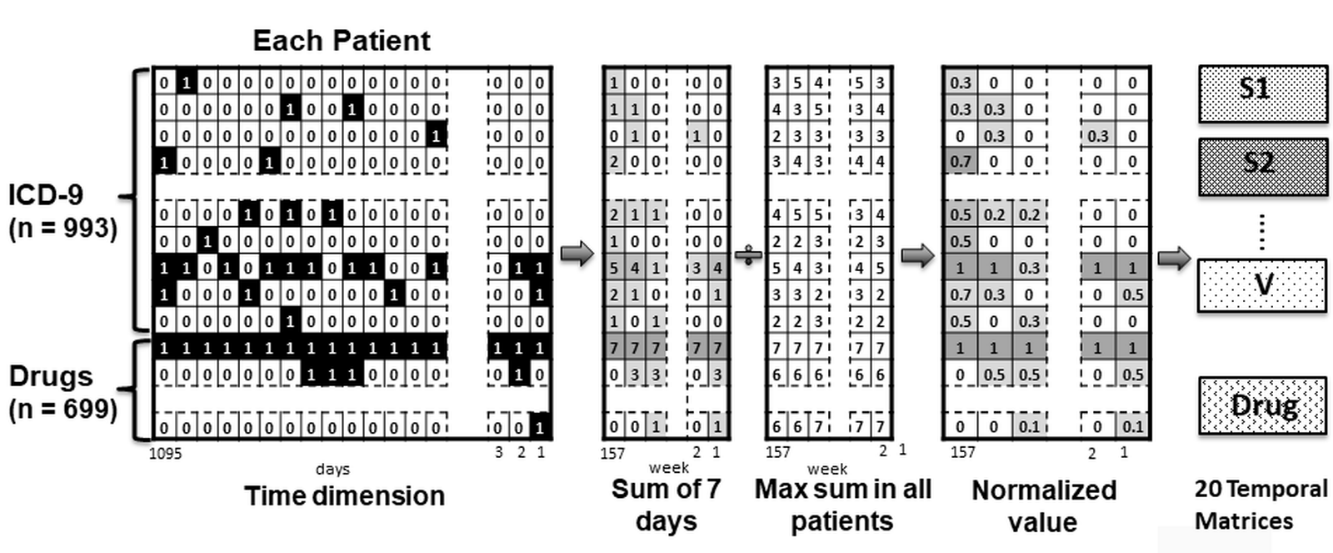
**

**Figure 1. Preprocess from matrix to 2O EHR image.**


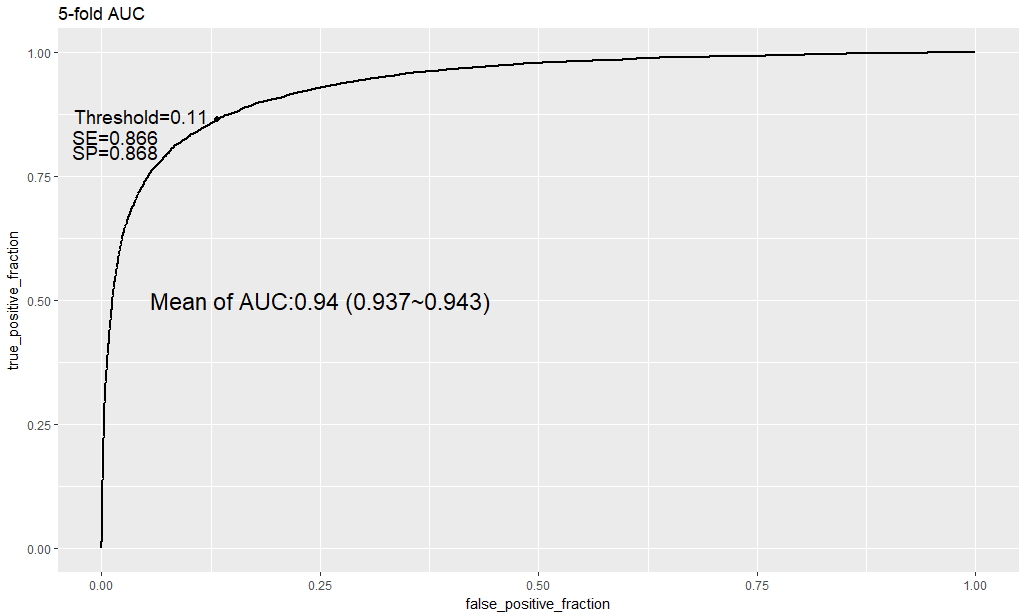


Figure S2: AUROC of predicting HCC one year ahead of time.

**Figure S3**: Prediction-Time vs. AUROC

Figure S4: Prediction performance with various variables
